# Arthritic Hand-Finger Movement Similarity Measurements: Tolerance Near Set Approach

**DOI:** 10.1155/2011/569898

**Published:** 2011-04-05

**Authors:** Christopher Henry, James F. Peters

**Affiliations:** Computational Intelligence Laboratory, Department of Electrical and Computer Engineering, University of Manitoba, Winnipeg, MB, Canada R3T 5V6

## Abstract

The problem considered in this paper is how to measure the degree of resemblance between nonarthritic and arthritic hand movements during rehabilitation exercise. The solution to this problem stems from recent work on a tolerance space view of digital images and the introduction of image resemblance measures. The motivation for this work is both to quantify and to visualize differences between hand-finger movements in an effort to provide clinicians and physicians with indications of the efficacy of the prescribed rehabilitation exercise. The more recent introduction of tolerance near sets has led to a useful approach for measuring the similarity of sets of objects and their application to the problem of classifying image sequences extracted from videos showing finger-hand movement during rehabilitation exercise. The approach to measuring the resemblance between hand movement images introduced in this paper is based on an application of the well-known Hausdorff distance measure and a tolerance nearness measure. The contribution of this paper is an approach to measuring as well as visualizing the degree of separation between images in arthritic and nonarthritic hand-finger motion videos captured during rehabilitation exercise.

## 1. Introduction

**Figure 1:**
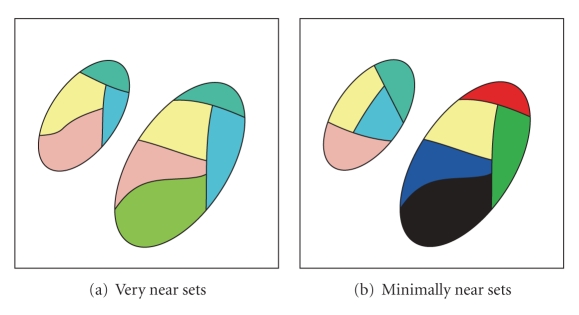
Sample near sets relative to color classes.

This paper presents an approach to quantifying and visualizing the degree of separation between images in arthritic and non-arthritic hand-finger motion videos captured during rehabilitation exercises. The proposed approach is based on tolerance near set theory. In this paper, a complete procedure for determining the degree of resemblance between non-arthritic and arthritic hand movements is presented. Measuring resemblances between hand motions during rehabilitation exercise has two main advantages: (i) apart from measurements of stiffness and pain before and after rehabilitation exercise, the separation as well as the degree of resemblance between what would be considered normal hand-finger motion and arthritic hand-finger motion can be measured (resemblance between sequences of non-arthritic and arthritic hand-finger movements are reported in this paper) and (ii) hand motion resemblance measurements provide a basis for assessing the efficacy of rehabilitation exercise regimes for arthritic patients. Videos made during hand-finger motion tracking that are part of a telerehabilitation system for automatic tracking and assessment of rehabilitation exercise by those with arthritis are a source of image sequences that are analyzed in this paper (see, e.g., [1]). The approach presented here can be used for assessment and comparison in problem domains that can be formulated in terms of a set of objects with descriptions represented by feature value vectors. A feature vector is an *n*-dimensional vector of numerical features representing an object description. Disjoint sets containing objects with similar descriptions are near sets. As an example of the degree of nearness between two sets, consider Figure 1 as two pairs of ovals containing colored segments. Each color in the figures corresponds to an equivalence class where all pixels in the class have matching descriptions, for example, pixels with matching colors. Thus, the ovals in Figure 1(a) are closer (more near) to each other in terms of their descriptions than the sets in Figure 1(b). Specifically, in comparing hand-finger movement images, image patches (collections of subimages with similar descriptions) provide information and reveal patterns of interest. The contribution of this paper is an approach to measuring as well as visualizing the degree of separation between images in arthritic and non-arthritic hand-finger motion videos captured during rehabilitation exercise.

This paper is organized as follows.  Section 2 presents related works to help establish a context for this research.  Section 3 gives a brief introduction to near set theory, Section 4 presents the image processing necessary to perform feature extraction on the hand images, Section 5 presents the algorithm used to generate the results presented in this paper, and finally Section 6 presents a discussion on the results.

## 2. Related Works

The hand-finger motion classification method reported in this paper is an outgrowth of earlier work on medical imaging [2, 3] and, in particular, on comparing hand movement image sequences [4]. The term *arthritis* is derived from the Greek words *arthron* (referring to joints) and the suffix *itis* (inflammation of). Interest in arthritis has not always been approached with as much fervor as other human ailments, particularly since the most common form (osteoarthritis) is not likely to be fatal [5]. However, human life expectancy has continued to improve, and, hence, an increase in arthritis cases is highly probable. Typically, with age there is a much greater likelihood of joints degrading and potentially wearing out. There are a a number of factors that lead to arthritis, for example, lifestyle, heredity, joint trauma, and even work-related, repetitive tasks [5]. Although the prognosis may not be fatal, quality of life for arthritis patients can be severely limited due to pain and disability. The resulting costs associated with health care for arthritis patients has become significant. Forbes published a list of the most expensive diseases and arthritis made the list in the USA, totaling 7.8 billion dollars of annual spending reported from 2002 [6]. As a result of reduced quality of life and the burden placed on health-care systems, continued research efforts are ongoing in drugs, joint replacements, intra-articular injections, and other experimental treatments of the disease [7].

A principal contribution of this paper is an application of near set theory in providing a basis for quantifying the extent that hand-finger motion images resemble each other. Near set theory has connections in topology [8], proximity spaces [9, 10], metric spaces [11], tolerance spaces [12, 13], and approach spaces [14, 15]. Near sets have proved to be useful in solving problems based on human perception [8] that arise in areas such as image analysis [2, 4, 14, 16], image processing [2, 4, 12, 13, 16–18], face recognition [19], ethology [20], image morphology, and segmentation evaluation [21, 22] as well as many engineering and science problems.

While the applications presented in this paper are based on the comparison of hand movement images, the proposed approach is suitable for investigation of problems formated in a similar manner. For example, Schubert et al. [23] presented a neural cell detection system to measure fluorescent lymphocytes in images of tissue sections. Their approach was to use a neural network, trained from a set of cell image patches, to determine if a pixel is the centre of one fluorescent cell. Each pixel was associated with a 6-dimensional feature vector generated by principal component analysis (PCA) on a 15 × 15 subimage centred on the pixel. Another example of a problem formated in a manner conducive to the proposed approach to discovering affinities in medical data is given by Yu et al. [24] in terms of a protein-protein interaction extraction from biomedical text. Given an abstract of an article containing instances of proteins, the system detects whether a relationship exists for each pair of proteins in the abstract. This problem is solved by using support vector machines, where each sentence containing a reference to proteins in a given abstract is considered an object and lexical and syntactic features are used to create a feature-value vector.

## 3. Tolerance Near Sets

Tolerance near sets are defined in the context of tolerance spaces. The term *tolerance space* was coined by Zeeman in 1961 in modelling visual perception with tolerances [25]. A tolerance space 〈*X*, ≃ 〉 consists of a set *X* and a binary relation ≃ on *X* (≃ ⊂*X* × *X*) that is reflexive (for all *x* ∈ *X*, *x* ≃ *x*, instead of (*x*, *y*) ∈ ≃ we write *x* ≃ *y*) and symmetric (for all *x*, *y* ∈ *X*, if *x* ≃ *y*, then *y* ≃ *x*) but transitivity of ≃ is not required. In this case, ≃ is called a *tolerance relation* (on *X*) or simply a *tolerance*.

All sets in near set theory consist of *perceptual objects*, defined as something that has its origin in the physical world. Moreover, all objects need to be described in some manner. This is accomplished by a *probe function*, a real-valued function representing a feature of a perceptual object [26]. Next, a *perceptual system *〈*O*, *𝔽*〉 consists of a nonempty set *O* of sample objects, and a non-empty set *𝔽* of real-valued functions *ϕ* ∈ *𝔽* such that *ϕ* : *O* → *ℜ* [8]. The elements of *O* are called perceptual objects and the functions in *𝔽* are called probe functions. The *description* of an object *x* ∈ *O* is a vector given by

(1) $$\begin{array}{c} \vec{\phi }\left(x\right)=\left(\phi_{1}\left(x\right),\phi_{2}\left(x\right),\ldots ,\phi_{i}\left(x\right),\ldots ,\phi_{l}\left(x\right)\right), \end{array}$$

where *l* is the length of the vector $\vec{\phi }$ and each *ϕ*_*i*_(*x*) in $\vec{\phi }(x)$ is a probe function value that is part of the description of the object *x* ∈ *O*. Keeping these concepts in mind, a perceptual tolerance relation can be described as follows.

**Definition 1 (perceptual tolerance relation [12, 13] see [2, 17] for applications))**

Let 〈*O*, *𝔽*〉 be a perceptual system and let *ε* ∈ ℝ. For every *ℬ* ⊆ *𝔽* a reflexive and symmetric tolerance relation ≅_*ℬ*,*ε*_ is defined as follows:

(2) $$\begin{array}{c} {≅}_{ℬ,\varepsilon }=\left\{\left(x,y\right)\in O\times O:\;{||\vec{\phi }\left(x\right)-\vec{\phi }\left(y\right)||}_{2}\leq \varepsilon \right\}. \end{array}$$

For notational convenience, this relation can be written as ≅_*ℬ*_ instead of ≅_*ℬ*,*ε*_ with the understanding that *ε* is inherent to the definition of the tolerance relation.

Definition 1 gives rise to two very useful types of sets, namely, a neighbourhood and a tolerance class. A *neighbourhood* of an object *x* ∈ *O* is defined as

(3) $$\begin{array}{c} N\left(x\right)=\left\{y\in O:x{≅}_{ℬ}y\right\}. \end{array}$$

**Figure 2:**
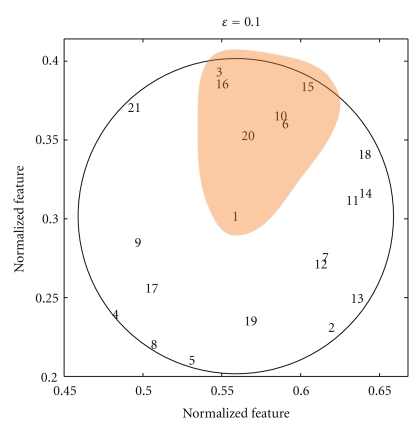
Example demonstrating the difference between a neighbourhood and a tolerance class in 2 dimensional feature space. The neighbourhood is all the objects within the circle, and the tolerance class is shown in orange.

An example of a neighbourhood in 2D feature space is given in Figure 2 where the position of all the objects is given by the numbers 1 to 21 and the neighbourhood is defined with respect to the object labelled 1. Notice that the distance between all the objects and object 1 is less than or equal to *ε* = 0.1 but that not all the pairs of objects in the neighbourhood of *x* satisfy the tolerance relation. In contrast, all the pairs of objects within a preclass must satisfy the tolerance relation. A set *X* ⊆ *O* is a *pre-class* when *x*≅_*ℬ*_ *y* for any pair *x*, *y* ∈ *X* [27]. A maximal pre-class with respect to inclusion is called a *tolerance class*. An example of a tolerance class is given in Figure 2 since no object can be added to the orange set and still satisfy the condition that any pair *x*, *y* ∈ *X* must be within *ε* of each other.

As mentioned above, we are interested in sets that have some objects that are similar to each other, where the term “similar” is quantified by the tolerance relation given in Definition 1. Thus, we introduce the following definition for tolerance near sets.

**Definition 2 (tolerance near set relation [12, 13])**

Let 〈*O*, *𝔽*〉 be a perceptual system, and let *X*, *Y* ⊆ *O*, *ε* ∈ ℝ. A set *X* is near to a set *Y* within the perceptual system $\langle O,𝔽\rangle \;(X{\underline{\underline{⋈}}}_{𝔽}Y)$ if and only if there exists *x* ∈ *X* and *y* ∈ *Y* and there is *ℬ* ⊆ *𝔽* such that *x* ≅_*ℬ*,*ε*_ *y*.

**Definition 3 (tolerance near sets [12, 13])**

Let 〈*O*, *𝔽*〉 be a perceptual system, and let *ε* ∈ ℝ, *ℬ* ⊆ *𝔽*. Further, let *X*, *Y* ⊆ *O* denote disjoint sets with coverings determined by the tolerance relation ≅_*ℬ*,*ε*_, and let *H*_≅_*ℬ*,*ε*__(*X*), *H*_≅_*ℬ*,*ε*__(*Y*) denote the set of tolerance classes for *X*, *Y*, respectively. Sets *X*, *Y* are *tolerance near sets* if and only if there are tolerance classes *A* ∈ *H*_≅_*ℬ*,*ε*__(*X*), *B* ∈ *H*_≅_*ℬ*,*ε*__(*Y*) such that $A{\underline{\underline{⋈}}}_{𝔽}B$.

As a practical example, consider an application in the area of image processing. Define an RGB image as *f* = {**p**_1_, **p**_2_,…, **p**_*T*_}, where **p**_*i*_ = (*c*, *r*, *R*, *G*, *B*)^T^, *c* ∈ [1, *M*], *r* ∈ [1, *N*], *R*, *G*, *B* ∈ [0,255], and *M*, *N*, respectively, denote the width and height of the image and *M* × *N* = *T*. Further, define a square subimage as *f*_*i*_ ⊂ *f* such that *f*_1_∩*f*_2_ ⋯ ∩*f*_*s*_ = *∅* and *f*_1_ ∪ *f*_2_ ⋯ ∪*f*_*s*_ = *f*, where *s* is the number of subimages in *f*. Next, *O* can be defined as the set of all subimages, that is, *O* = {*f*_1_,…, *f*_*s*_}, and *𝔽* is a set of functions that operate on images. Then the sets *X*, *Y* ∈ *O* are perceptually near each other if there are *x* ∈ *X* (i.e., subimages from *X*) and *y* ∈ *Y* (i.e., subimages from *Y*) and there is *ℬ* ⊆ *𝔽* such that *x* ≅_*ℬ*_ *y*. This would be the case when there are two or more subimages that have similar descriptions using the probe functions in *ℬ*.

Definition 2 provides a means of determining whether two sets of perceptual objects are near each other. Suppose, however, that we want to consider the problem of comparing objects in tolerance near sets (such as sets created by separate images) and measure the degree of similarity between the two sets. This problem is of interest because its solution provides a formal basis for measuring the resemblance of sets of objects that are described by feature value vectors and has many applications, such as the problem of measuring image resemblance presented in this paper. In other words, a method for determining the degree in which two tolerance near sets are similar is needed. Let *X* and *Y* be two disjoint sets, and let *Z* = *X* ∪ *Y*. Then a nearness measure [2, 28, 29] between two sets is given by

(4) $$\begin{array}{cc} \text{tN}{\text{M}}_{{≅}_{ℬ,\varepsilon }}\left(X,Y\right) & ={\left(\underset{C\in H_{{≅}_{ℬ,\varepsilon }}\left(Z\right)}{\sum }\left|C\right|\right)}^{-1}\\ & \;\cdot \underset{C\in H_{{≅}_{ℬ,\varepsilon }}(Z)}{\sum }\left|C\right|\frac{\min\;\;\left(\left|C\cap X\right|,\left|C\cap Y\right|\right)}{\max\;\;\left(\left|C\cap X\right|,\left|C\cap Y\right|\right)}. \end{array}$$

The idea behind (4) is that similar sets should produce tolerance classes that are evenly divided between *X* and *Y*. This is measured by counting the number of objects that belong to sets *X* and *Y* for each tolerance class and then comparing these counts as a proper fraction. Then, the measure is simply a weighted average of all the fractions. A weighted average was selected to give preference to larger tolerance classes with the idea that a larger tolerance class contains more perceptually relevant information.

**Figure 3:**
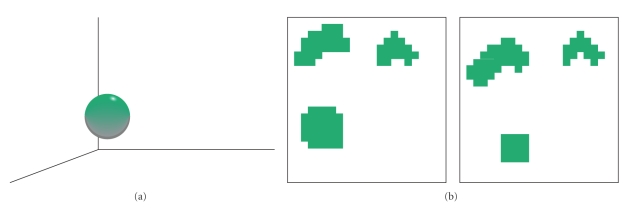
Example relating tolerance class objects to their coordinates within a pair of images. (a) Tolerance class in 3 dimensional feature space. (b) Image coordinates of objects belonging to the tolerance class in (a).

Calculating the proper fraction for a single tolerance class *C* is shown graphically in Figure 3 using the example given above concerning subimages.  Figure 3(a) gives a sample tolerance class in 3D feature space, while Figure 3(b) shows the position of the subimages in the images (i.e., sets *X* and *Y*) that belong to the tolerance class in Figure 3(a). Observe that a tolerance class in feature space can be distributed throughout the images and that tNM would compare the number of objects from the tolerance class in set *X* to the number of objects from the tolerance class in set *Y*. In this case, the ratio would be close to 1 because the number of objects in both sets *X* and *Y* are roughly the same.

## 4. Segmenting Hand Motion Images and Feature Extraction

**Figure 4:**
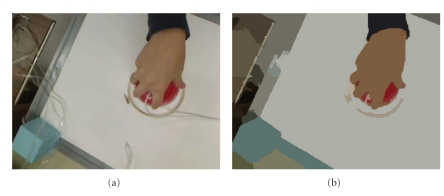
Example of the mean shift segmentation algorithm [31]. (a) Sample image from database used in this article. (b) Segmentation of (a) using the EDISON system [32].

Recall that the focus of this paper is to present an application of the tolerance near set approach by way of comparing the hand movements of an arthritic patient with normal hand movements during rehabilitation exercises. Digital images obtained from video captured during the exercises are used to make the comparison (see, e.g., [30]). As a result, a brief presentation of the image segmentation and feature extraction methods used in the reported experiments are presented in this section. Measuring the resemblance of hand motion images is made possible by segmenting the images. Segmenting a digital image is a separation of image regions that are nonoverlapping and is important in this work since it facilitates separation of image background from the portion of a hand in an image (see, e.g., Figure 4). 

### 4.1. Mean Shift Segmentation Algorithm

The mean shift algorithm (introduced in [31]) segments an image using kernel density estimation, a nonparametric technique for estimating the distribution of a random variable. Nonparametric techniques are characterized by their lack of assumptions about the distributions and differ from parametric techniques which assume a given distribution and then estimate parameters which describe the density, like mean or variance [34]. The estimate of the distribution at a point **x** is calculated from the number of observations within a volume in *d*-dimensional space centred on **x** and a kernel that weights the importance of the observations [34]. The segmentations used in this paper were created using an implementation of the mean shift segmentation algorithm called EDISON [32], a system for which both the source code and binaries are freely available online. A sample segmentation produced by the EDISON system is given in Figure 4.

### 4.2. Multiscale Edge Detection

**Figure 5:**
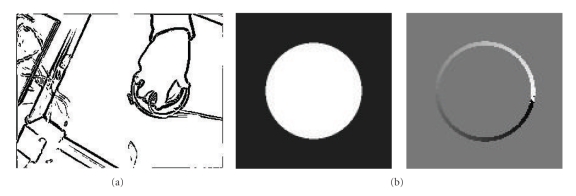
(a) Example demonstrating implementation of Mallat's multiscale edge detection method [33]. (b) Example of finding edge orientation using the same method. White represents 0 radians and black 2*π* radians.

Mallat's multiscale edge detection method uses Wavelet theory to find edges in an image [33]. Edges are located at points of sharp variation in pixel intensity identified by calculating the gradient of a smoothed image (i.e., an image that has been blurred). Then, edge pixels are defined as those that have locally maximal gradient magnitudes in the direction of the gradient. Examples of our own implementation of Mallat's edge detection and edge orientation methods are given in Figure 5.

### 4.3. Feature Extraction

**Figure 6:**
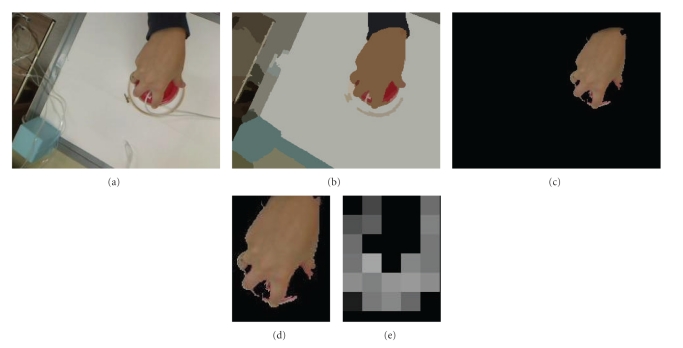
Figure showing preprocesing required to create tolerance classes and calculate tNM. (a) Original image. (b) Segmented image. (c) Hand segment only. (d) Cropped image to eliminate useless background. (e) Final image used to obtain tolerance classes. Each square represents an object where the colour (except black) represents the average orientation of a line segment within that subimage.

An example of the type of images obtained directly from the video is given in Figure 6(a). These images needed to be further processed to remove the common background (e.g., all the images contain the white desktop, the square blue sensor, etc.) that would produce results indicating that all the images were similar. Therefore, the mean shift segmentation algorithm was used to create a segment containing only the hand in each image. The resultant segmented image is given in Figure 6(b) where pixels with similar colour are now grouped together into segments. The next step was to use the segment representing the hand as a mask to separate the hand from the original image (given in Figure 6(c)). Next, notice the absence of the majority of the black background (representing the masked pixels in the original image) in Figure 6(d). Each image was cropped to an image containing only the hand because the output of probe functions on the black background would be the same for each image.

Next, perceptual objects are created in the same manner as the example given in Section 3. Specifically, each image was divided into square subimages such that no subimage overlapped, where each subimage represents an object in the near set sense and a probe function is then any function that can operate on images. In this case, we used only one probe function, namely, the average orientation of lines within a subimage. For example, the orientation can be determined (using the process given in Section 4.2) for each pixel considered part of a line detected in an image. Then, the probe function takes an average of all the orientations for pixels belonging to edges within a specific subimage. An example of the output of this probe function is given in Figure 6(d).

## 5. Tolerance Class Algorithm

The practical application of the nearness measure, tNM, rests on the ability to efficiently find all the classes for a set *Z* = *X* ∪ *Y*. In the case where *ε* = 0, the process is straightforward, that is, the first object is assigned to a tolerance class, then the description of each subsequent object is compared to objects in each existing tolerance class. If a given object's description does not match any of the descriptions of the existing tolerance classes, then a new class is created. Thus, the algorithm runtime ranges from order *O*(|*Z*|^2^) in the worst case, which occurs when none of the object descriptions match, to *O*(|*Z*|), which occurs when all the object descriptions are equivalent. In practice, the runtime is somewhere between these two extremes.

The approach to finding tolerance classes in the case where *ε* ≠ 0 is based on the observations presented in the following Propositions.

**Proposition 1**

Given a tolerance space 〈*X*, ≅_*ℬ*,*ε*_〉, all tolerance classes containing *x* ∈ *X* are subsets of neighbourhood *N*(*x*).

**Proof**

Given a tolerance space 〈*X*, ≅_*ℬ*,*ε*_〉 and tolerance class *A* ⊂ ≅_*ℬ*,*ε*_, then (*x*, *y*) ∈ ≅_*ℬ*,*ε*_ for every *x*, *y* ∈ *A*. Let *N*_≅_*ℬ*,*ε*__(*x*) be a neighbourhood of *x* ∈ *X* and assume that *x* ∈ *A*. For *y* ∈ *A*, (*x*, *y*) ∈ ≅_*ℬ*,*ε*_. Hence, *A* ⊂ *N*_≅_*ℬ*,*ε*__(*x*). As a result, *N*_≅_*ℬ*,*ε*__(*x*) is superset of all tolerance classes containing *x*.

**Proposition 2**

Let *z*_1_,…, *z*_*n*_ ∈ *Z* be a succession of objects, called query points, such that *z*_*n*_ ∈ *N*(*z*_*n*−1_)\*z*_*n*−1_, *N*(*z*_*n*_) ⊆ *N*(*z*_*n*−1_)\*z*_*n*−1_ ⊆ ⋯ ⊆ *N*(*z*_1_)\*z*_1_, and define *N*(*z*_0_)\*z*_0_ as the original set of objects (i.e., *N*(*z*_0_)\*z*_0_ = *Z*). In other words, the series of query points, *z*_1_,…, *z*_*n*_ ∈ *Z*, is selected such that each subsequent object *z*_*n*_ (where *z*_*n*_ ≠ *z*_*n*−1_) is obtained from the neighbourhood *N*(*z*_*n*−1_), that is created only using objects from the previous neighbourhood. Then, under these conditions, the set {*z*_1_,…, *z*_*n*_} is a pre-class.

**Proof**

For *n* ≥ 2, let *S*(*n*) be the statement that {*z*_1_,…, *z*_*n*_} is a pre-class given the conditions in Proposition 2.

**Base Step (*n* = 2)**

Let *z*_1_ ∈ *Z* be the first query point, and let *N*(*z*_1_) be the first neighbourhood. Next, let *z*_2_ represent the next query object. Since *z*_2_ must come from *N*(*z*_1_) and all objects in *x* ∈ *N*(*z*_1_) satisfy the tolerance relation *z*_1_≅_*ℬ*,*ϵ*_ *x*, *S*(2) holds.

**Inductive Step**

Fix some *k* ≥ 2 and suppose that the inductive hypothesis holds, that is, {*z*_1_,…, *z*_*k*_} is a pre-class, and choose *z*_*k*+1_ from *N*(*z*_*k*_)\*z*_*k*_. Since *N*(*z*_*k*_) ⊆ *N*(*z*_*k*−1_)\*z*_*k*−1_ ⊆ ⋯ ⊆ *N*(*z*_1_)\*z*_1_, *z*_*k*+1_ must satisfy the perceptual tolerance relation with all the objects in {*z*_1_,…, *z*_*k*_}. Consequently, {*z*_1_,…, *z*_*k*+1_} is also a pre-class.

Therefore, by MI, *S*(*n*) is true for all *n* ≥ 2.

**Corollary 1**

Let *z*_1_,…, *z*_*n*_ ∈ *Z* be a succession of objects, called query points, such that *z*_*n*_ ∈ *N*(*z*_*n*−1_)\*z*_*n*−1_, *N*(*z*_*n*_) ⊆ *N*(*z*_*n*−1_)\*z*_*n*−1_ ⊆ ⋯ ⊆ *N*(*z*_1_)\*z*_1_, and define *N*(*z*_0_)\*z*_0_ as the original set of objects (i.e., *N*(*z*_0_)\*z*_0_ = *Z*). In other words, the series of query points, *z*_1_,…, *z*_*n*_ ∈ *Z*, is selected such that each subsequent object *z*_*n*_ (where *z*_*n*_ ≠ *z*_*n*−1_) is obtained from the neighbourhood *N*(*z*_*n*−1_) that is created only using objects from the previous neighbourhood. Then, under these conditions, the set {*z*_1_,…, *z*_*n*_} is a tolerance class if |*N*(*z*_*n*_)| = 1.

**Proof**

Since the cardinality of *N*(*z*_1_) is finite for any practical application and the conditions given in Proposition 2 dictate that each successive neighbourhood will be smaller than the last, there is an *n* such that |*N*(*z*_*n*_)| = 1. By Proposition 2 the series of query points {*z*_1_,…, *z*_*n*_} is a pre-class, and by Proposition 1 there are no other objects that can be added to the class {*z*_1_,…, *z*_*n*_}. As a result, this pre-class is maximal with respect to inclusion and by definition is called a tolerance class.

**Figure 7:**
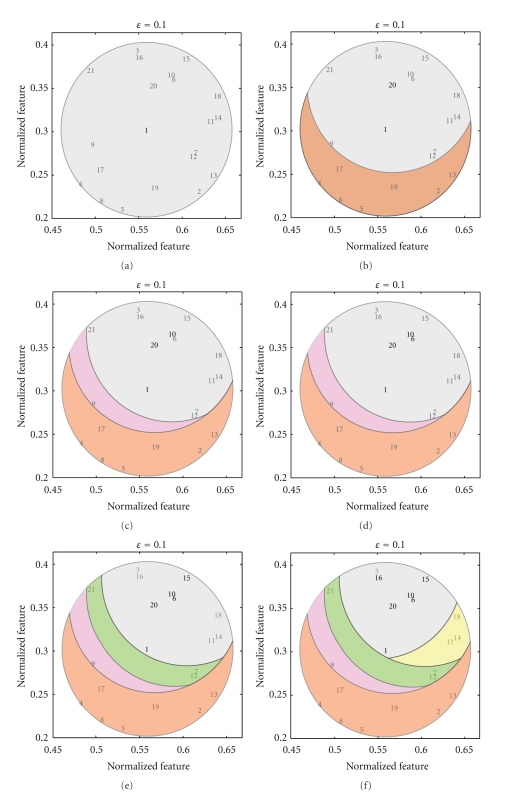
Visualization of Propositions 1 and 2 and Corollary 1. (a) *N*(1), (b) *N*(20), created using only objects from *N*(1), (c) *N*(10), created using only objects from *N*(20) (which was created using only objects from *N*(10)), (d) *N*(6), again created using only objects from *N*(10), and so forth, (e) *N*(15), and (f) *N*(16).

The above observations are visualized in Figure 7 using the example first introduced in Figure 2. Starting with the the proof of Proposition 2, a visual example of the base step is given in Figures 7(a) and 7(b). In this case, only the first 21 objects of *Z* are shown, where *z*_1_ is the object labelled 1 and *N*(*z*_1_) is the circle containing the objects {1,…, 21}. Next, according to Proposition 2, another query point *z*_2_ ∈ *N*(*z*_1_)\*z*_1_ is selected (i.e., *z*_2_ can be any object in *N*(*z*_1_) except *z*_1_). Here, *z*_2_ = 20 is selected because it is the next object closest to *z*_1_. Since *z*_1_≅_*ℬ*,*ϵ*_ *z*_2_, the class {*z*_1_, *z*_2_} is a pre-class. Also, note that Figure 7(b) also gives an example of *N*(*z*_2_) ⊂ *N*(*z*_1_) as the area shaded grey, and the area shaded red is the part of *N*(*z*_1_) that does not satisfy the tolerance relation with *z*_2_. Continuing on, an example of the inductive step from the proof of Proposition 2 is given in Figure 7(e). In this case, there are *k* = 5 objects and {*z*_1_,…, *z*_5_} = {1,20,10,6, 15}. The area shaded grey represents *N*(*z*_5_)\*z*_5_ ⊂ ,…, ⊂*N*(*z*_1_)\*z*_1_ along with the query points {*z*_1_,…, *z*_5_}(according to the conditions given in Proposition 2 queries points are not included in subsequent neighbourhoods), and the other shaded areas represent the parts of successive neighbourhoods that no longer satisfy the tolerance relation with every query point. For instance, all the colours except red are in *N*(20), and all the colours except red and purple are in *N*(10) and *N*(6). Notice that all the objects in the grey area satisfy the tolerance with all the query points but that the grey area does not represent a pre-class. Moreover, any new query point selected from *N*(*z*_5_)\*z*_5_ = {16,18,3, 14,11} will also satisfy the tolerance relation with all the query points {*z*_1_,…, *z*_5_}. Finally, Figure 7(f) demonstrates the idea behind Corollary 1. In this figure, the area shaded grey represents the neighbourhood of *z*_7_ = 3 along with all previous query points. Observe that (besides query points) the shaded area only contains one object, namely, *z*_7_. Also, note that there are no more objects that will satisfy the tolerance relation with all the objects in the shaded area. As a result, the set {*z*_1_,…, *z*_7_} is a tolerance class. 

Using Propositions 1 and 2 and Corollary 1, the following algorithm gives the pseudocode for an approach for finding all the tolerance classes on a set of objects *Z*. The general concept of the algorithm is, for a given object *z* ∈ *Z*, to recursively find all the tolerance classes containing *z*. The first step, based on Proposition 1, is to set *z* as a query point and to find the neighbourhood *N*(*z*). Next, consider the nearest neighbour of *z* from the neighbourhood *N*(*z*) as a query point and find its neighbourhood only considering objects in *N*(*z*). Continue this process until the result of a query produces a neighbourhood with cardinality 1. (The result of a query will always be at least 1 since the tolerance relation is reflexive.)

Lastly, the series of query points becomes the tolerance class.

**Algorithm 1 (see [28])**

1. Take an element *z* ∈ *Z* and find *N*_≅_*ℬ*,*ε*__(*z*).
2. Add *z* to a new tolerance class *C*. Select an object *z*′ ∈ *N*_≅_*ℬ*,*ε*__(*z*) such that *z*′ ≠ *z*.
3. Add *z*′ to *C*. Find neighbourhood *N*_≅_*ℬ*,*ε*__(*z*′) using only objects from *N*_≅_*ℬ*,*ε*__(*z*). Do not include *z* in *N*_≅_*ℬ*,*ε*__(*z*′). Select a new object *z*′′ ∈ *N*_≅_*ℬ*,*ε*__(*z*′) such that *z*′′ ≠ *z*′. Relabel *z* ← *z*′, *z*′ ← *z*′′ and *N*_≅_*ℬ*,*ε*__(*z*) ← *N*__≅_*ℬ*,*ε*___(*z*′).
4. Repeat step 3 until a neighbourhood of only 1 element is produced. When this occurs, add the last element to *C* and then add *C* to H_*ℬ*_^*ε*^(*Z*).
5. Perform step 2 (and subsequent steps) until each object in *N*_≅_*ℬ*,*ε*__(*z*) has been selected at the level of step 2.
6. Perform step 1 (and subsequent steps) for each object in *Z*.
7. Delete any duplicate classes.

Finally, note the following. We used an added heuristic for step 2 to reduce the computation time of the algorithm. Namely, an object from *N*_≅_*ℬ*,*ε*__(*z*) can only be selected as *z*′ in step 2 if it has not already been added to a tolerance class created from *N*_≅_*ℬ*,*ε*__(*z*) (i.e., this rule is reset each time step 1 is visited). In addition, the Fast Library for Approximate Nearest Neighbours [35, 36] was used to find all the neighbourhoods in this algorithm.

The tolerance class originally given in Figure 2 was produced using this algorithm, and the intermediate steps of this algorithm are visualized in Figure 7. To begin with, Figure 7(a) represents Step 1 of the algorithm with *z* = 1. Step 2 is given in Figure 7(b), where *z*′ = 20. Steps 3 and 4 are given in Figures 7(c)–7(f). Observe that in Figure 7(f) |*N*_≅_*ℬ*,*ε*__(3)| = 1 since all the other bold objects in the grey area have been added to *C*, and, as such, are not allowed to be included in subsequent neighbourhoods. Step 5 can be explained as follows. Figure 7 shows the sequence of steps for selecting *z* = 20 (the closest object to 1) at the level of Step 2. Hence, Step 5 states that each object in the neighbourhood of 1 (except 1 itself) should be selected at Step 2. Moreover, the heuristic given after the algorithm states that any object added to a tolerance class derived from the neighbourhood of 1 should not be considered at Step 2. As a result, in this example, the objects {3,6, 10,15,16} should not be considered again at Step 2 for finding tolerance classes derived from the neighbourhood of object 1. Lastly, note that Step 1 must be performed for all objects in *Z*.

Finally, this section is concluded by mentioning a few observations about the algorithm. The runtime of the algorithm in the worst case is *O*(|*Z*|^2^ *T*), where *T* is the complexity of finding an object's neighbourhood among the other |*Z* | − 1 objects. However, it should be noted that the algorithm is rarely run on the worst case data. The worst case suggests that either the epsilon value is much too large or that the data is so clustered that, from a perceptual point of view, every pair of objects in the set resembles each other. In either case, the data is not interesting from a nearness measure or image correspondence perspective. The runtime on typical data is of order *O*(|*Z* | *cT*), where *c* ≤ |*Z*| is a constant based on the object *z* ∈ *Z* that has the largest neighbourhood. Lastly, this algorithm lends itself to parallel processing techniques, and the results in this paper were also obtained using multithreading on a quad core processor. A comparison of two images used to generate the results in this paper using our implementation was on the order of 0.2 sec.

## 6. Results

The goal of this paper is to present an application of the tolerance near set approach by way of comparing the hand movements of an arthritic patient with normal hand movements during rehabilitation exercises. Consequently, this section presents results of comparing images from three patients, two of which do not have arthritis, using the tolerance near set approach. As mentioned, the images were obtained from a video taken during a rehabilitation exercise (see, e.g. [30]). This section presents the selection of parameters used to obtain the results and ends with a look at a comparison of tNM with an existing measure called the Hausdorff distance.

### 6.1. Selection of Epsilon

For normalized feature values, the largest distance between two objects occurs when one object has a feature vector (object description) of all zeros and the other has a feature vector of all ones. As a result, *ε* is in the interval $\left[0,\sqrt{l}\right]$, where *l* is the length of the feature vectors. In any given application, there is always an optimal *ε* when performing experiments using the perceptual tolerance relation. For instance, a value of *ε* = 0 produces little or no pairs of objects that satisfy the perceptual tolerance relation, and a value of $\varepsilon =\sqrt{l}$ means that all pairs of objects satisfy the tolerance relation. Consequently, *ε* should be selected such that the objects that are relatively (Here, distance of “objects that are relatively close” will be determined by the application.) close in feature space satisfy the tolerance relation, and the rest of the pairs of objects do not. The selection of *ε* is straightforward when a metric is available for measuring the success of the experiment. In this instance, the value of *ε* is selected based on the best result of the evaluation metric, where a plot of *ε* versus the metric usually resembles an inverted parabola. Fortunately, in this case, precision versus recall plots, defined in the context of image retrieval, can be used to evaluate the effectiveness of *ε*.

To demonstrate the selection of *ε*, the database of hand-finger movement images from three patients is used. One of the patients has rheumatoid arthritis, while the other two do not. Here, the goal is to perform content-based image retrieval and separate the images into three categories, one for each patient. An image belonging to one of the three patients is used as a query image, and then the images are ranked in descending order based on the value of tNM with the query image. For example, the database of images contains 98 images, of which 30 are from the patient with arthritis, and, respectively, 39 and 29 of them are from two patients without arthritis. Then, each image is in turn selected as the query image, and a value of tNM between the query image and every other image in the database is determined. Subsequently, a tolerance *ε* can be selected based on the number of images that are retrieved from the same category as the query image before a false negative occurs (i.e., before an image from a category other than the query image occurs).

**Figure 8:**
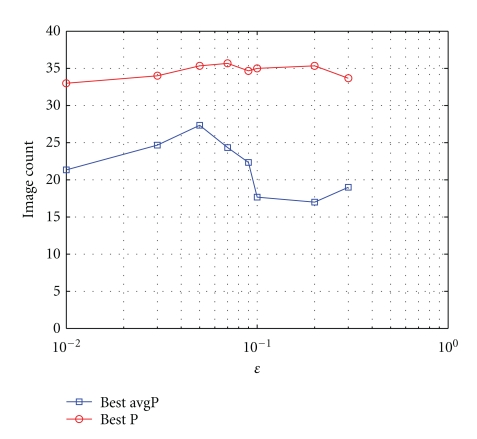
Plot giving the number of images retrieved before the precision falls below 90%.

Using this approach, Figure 8 contains a plot showing the number of images retrieved before the precision dropped below 90% for a given value of *ε*. The image (out of all possible 98 images) that produced the best query results is given in red, and the average is given in blue. Notice that the best results in the average case occur with tolerance *ε* = 0.05, which is close to the *ε* = 0.07 in the best case. This plot suggests that retrieval of images in this database benefits from a slight easying of the equivalence condition, but not much.

**Figure 9:**
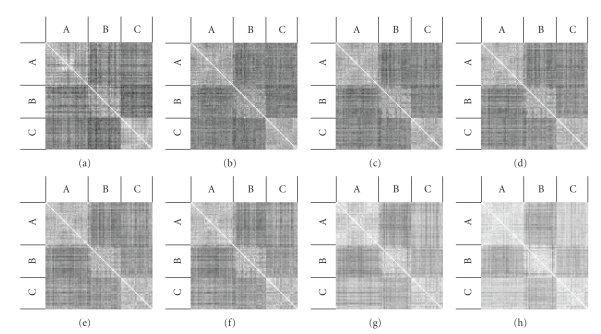
Images of nearness measure obtained from comparing the 98 images from three subjects to each other. (a)–(h) Visualization of nearness measure using *ε* ∈ {0.01,0.03,0.05,0.07,0.09,0.1,0.2,0.3}. Patients B has arthritis, while A and C do not.

Verifying the validity of selecting *ε* in this manner can be accomplished both by the visualization of the nearness measure for all pairs of images in the experiment and by observing the precision recall plots directly. First, an image can be created where the height and width are equal to the number of images in the database, each pixel corresponds to the value of tNM generated from the comparison of two images, and the colours black and white correspond to a nearness measure of 0 and 1, respectively. For example, an image of size 98 × 98 can be created like the one in Figure 9(a) where patient B is the one with arthritis, and each pixel corresponds to the nearness measure between two pairs of images in the database. Notice that a checkered pattern is formed with a white line down the diagonal. The white line corresponds to the comparison of an image with itself in the database, naturally producing a nearness measure of 1. Moreover, the lightest squares in the image are formed from comparisons between images from the same patient, and the darkest squares are formed from comparisons between the arthritis and healthy images. Also notice that the boundaries in Figures 9(c) and 9(d) are more distinct than for images created by other values of *ε* suggesting that *ε* = 0.05 or *ε* = 0.07 is the right choice of *ε*. Similarly, the square corresponding to patient C has crisp boundaries in Figures 9(a) and 9(h) and is also the brightest area of the figure, suggesting that a value of *ε* = 0.3 would also be a good choice for images belonging to patient C.

**Figure 10:**
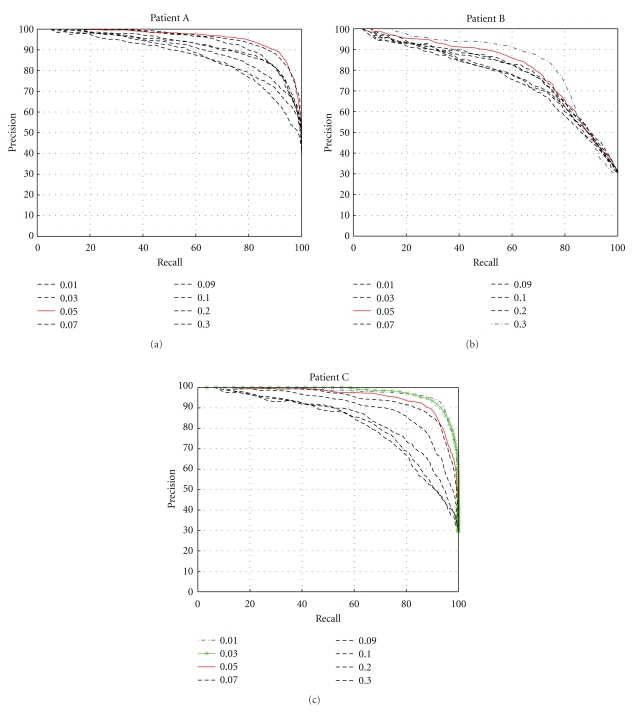
Plots showing the average precision recall plots for patients A–C.

Next, Figure 10 gives plots of the average precision versus recall for each patient. These plots were created by fixing a value of *ε* and calculating precision versus recall for each image belonging to a patient. Then, the average of all the precision/recall values for a specific value of *ε* are added to the plot for each patient. The results for selecting *ε* = 0.05 are given in red, and, in the case of patients B and C, the choice of *ε* that produced a better result than *ε* = 0.05 is also highlighted.

### 6.2. Hausdorff Distance

This section introduces an additional measure for determining the degree that near sets resemble each other. The Hausdorff distance is used to measure the distance between sets in a metric space [37] (see [38] for English translation) and is defined as

(5) $$\begin{array}{c} d_{H}\left(X,Y\right)=\max\;\;\left\{\;\underset{x\in X}{\sup\;\;}\;\underset{y\in Y}{\inf\;\;}\;d\left(x,y\right),\;\underset{y\in Y}{\sup\;\;}\;\underset{x\in X}{\inf\;\;}\;d\left(x,y\right)\right\}, \end{array}$$

where sup  and inf  refer to the supremum and infimum and *d*(*x*, *y*) is the distance metric (in this case it is the *l* ^2^ norm). The distance is calculated by considering the distance from a single element in a set *X* to every element of set *Y*, and the shortest distance is selected as the infimum. This process is repeated for every *x* ∈ *X*, and the largest distance (supremum) is selected as the Hausdorff distance of the set *X* to the set *Y*. This process is then repeated for the set *Y* because the two distances will not necessarily be the same. Keeping this in mind, the measure tHD [29] is defined as

(6) $$\begin{array}{cc} {\text{tHD}}_{{≅}_{ℬ,\varepsilon }}\left(X,Y\right) & ={\left(\underset{C\in H_{{≅}_{ℬ,\varepsilon }}\left(Z\right)}{\sum }\left|C\right|\right)}^{-1}\\ & \;\cdot \underset{C\in H_{{≅}_{ℬ,\varepsilon }}\left(Z\right)}{\sum }\left|C\right|\left(\sqrt{l}-\;d_{H}\left(C\cap X,C\cap Y\right)\right). \end{array}$$

Observe that low values of the Hausdorff distance correspond to a higher degree of resemblance than larger distances. Consequently, the distance is subtracted from the largest distance $\sqrt{l}$. The Hausdorff distance is a natural choice for comparison with the tNM nearness measure because it measures the distance between sets in a metric space. Recall that tolerance classes are sets of objects with descriptions in *l*-dimensional feature space. The nearness measure evaluates the split of a tolerance class between sets *X* and *Y*, where the idea is that a tolerance class should be evenly divided between *X* and *Y*, if the two sets are similar (or the same). In contrast, the Hausdorff distance measures the distance between two sets. Here the distance being measured is between the portions of a tolerance class in sets *X* and *Y*. Thus, two different measures can be used on the same data, namely, the tolerance classes obtained from the union of *X* and *Y*. 

### 6.3. Comparison between Hausdorff and tNM Measures

**Figure 11:**
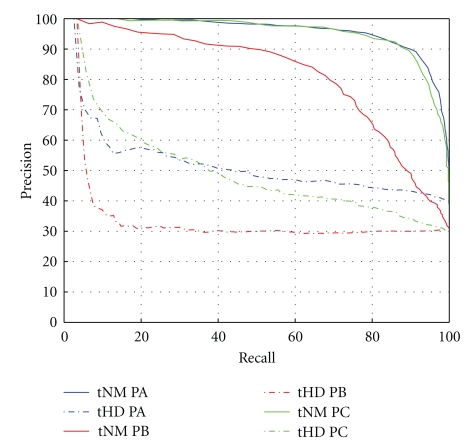
Plot of precision recall values for nearness measure and Hausdorff distance with *ε* = 0.05.

**Figure 12:**
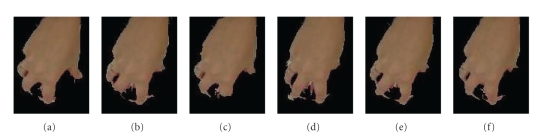
Results of image retrieval using a randomly selected query image from patient A. (a) Query image, and (b)–(f) images producing the top five nearness measures.

**Figure 13:**
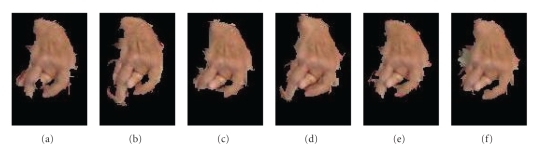
Results of image retrieval using a randomly selected query image from patient B. (a) Query image, and (b)–(f) images producing the top five nearness measures.

**Figure 14:**
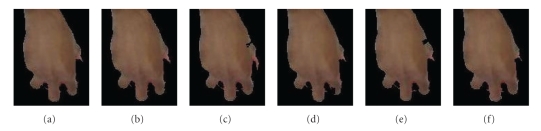
Results of image retrieval using a randomly selected query image from patient C. (a) Query image, and (b)–(f) images producing the top five nearness measures.

Next, Figure 11 contains the comparison of the two measures. The precision recall data for the Hausdorff distance was generated with *ε* = 0.5. Again, the data was obtained by taking an average of all the precision (and recall) values for each image belonging to a particular patient. Notice that the nearness measure performs better, that is, the precision recall plot is closer to ideal for all three patients using the nearness measure. The reason is that the performance of the Hausdorff distance is poor for low values of *ε*, since, as tolerance classes start to become equivalence classes (i.e., as *ε* → 0), the Hausdorff distance approaches 0 as well. Thus, if each tolerance class is close to an equivalence class, the resulting distance will be zero and consequently the measure will produce a value near to 1, even if the images are not alike. In contrast, as *ε* increases, the members of classes tend to become separated in feature space, and, as a result, only classes with objects that have objects in *X* that are close to objects in *Y* will produce a distance close to zero. What does this imply? If for a larger value of *ε*, relatively speaking, the set of objects *Z* = *X* ∪ *Y* still produces tolerance classes with objects that are tightly clustered, then this measure will produce a high measure value. Notice that this distinction is only made possible if *ε* is relaxed. Otherwise, all tolerance classes will be tightly clustered. Finally, Figures 12, 13, and 14 show the top five retrieved results for randomly selected query image of each category. Observe that the results all belong to the right category, which is as expected based on the precision recall plots.

## 7. Concluding Remarks

This paper focuses on the analysis, classification, and visualization of hand-finger movement images extracted from videos made during rehabilitation exercise sessions for osteoarthritic clients. This work stems from the need to provide healthcare providers and clients with resemblance measures, and hand-figure movement image analysis and visualization of the results of content-based image retrieval. Two forms of image resemblance measures are considered, the Hausdorff distance tHD and a tolerance near set resemblance measure tNM. The results reported in this paper suggest that the tNM measure is more accurate than the well-known Hausdorff distance measure. In addition, two forms of visualization of a tolerance space view of hand-finger motion during rehabilitation exercise are presented. In addition to watching videos of rehabilitation therapy sessions, it is now possible to compare arthritic and non-arthritic hand movements in entirely different ways, that is, comparisons can be made using checkerboard grids and precision recall plots. A checkerboard greyscale grid like the one in Figure 9 gives a qualitative view of hand-figure movement images extracted from rehabilitation exercise videos. That is, the greater the contrast between the grey areas reflecting arthritic and non-arthritic hand-finger movements, the greater the disparity between client hand movements. By contrast, precision recall plots like the ones in Figure 10 give a quantitative comparison of the results of different tolerances in measuring resemblance between hand-finger movement images.
